# Multi-omics analyses provide insights into the sulfur metabolism of a novel deep-sea sulfate-reducing bacterium

**DOI:** 10.1016/j.isci.2024.110095

**Published:** 2024-05-23

**Authors:** Chong Wang, Rikuan Zheng, Chaomin Sun

**Affiliations:** 1 CAS and Shandong Province Key Laboratory of Experimental Marine Biology & Center of Deep Sea Research, Institute of Oceanology, Chinese Academy of Sciences, Qingdao, China; 2 Laboratory for Marine Biology and Biotechnology, Qingdao National Laboratory for Marine Science and Technology, Qingdao 266071, China; 3 Center of Ocean Mega-Science, Chinese Academy of Sciences, Qingdao 266071, China; 4 College of Earth Science, University of Chinese Academy of Sciences, Beijing 100049, China

**Keywords:** Microbial metabolism, Microbiology, Omics

## Abstract

Sulfate-reducing bacteria (SRB) are ubiquitously distributed across various biospheres and play key roles in global sulfur and carbon cycles. However, few deep-sea SRB have been cultivated and studied *in situ*, limiting our understanding of the true metabolism of deep-sea SRB. Here, we firstly clarified the high abundance of SRB in deep-sea sediments and successfully isolated a sulfate-reducing bacterium (zrk46) from a cold seep sediment. Our genomic, physiological, and phylogenetic analyses indicate that strain zrk46 is a novel species, which we propose as *Pseudodesulfovibrio serpens*. We found that supplementation with sulfate, thiosulfate, or sulfite promoted strain zrk46 growth by facilitating energy production through the dissimilatory sulfate reduction, which was coupled to the oxidation of organic matter in both laboratory and deep-sea conditions. Moreover, *in situ* metatranscriptomic results confirmed that other deep-sea SRB also performed the dissimilatory sulfate reduction, strongly suggesting that SRB may play undocumented roles in deep-sea sulfur cycling.

## Highlights

• Clarified the high abundance of SRB in deep-sea cold seep sediments
• Isolated a novel SRB from a deep-sea cold seep sediment
• The novel SRB performed DSR coupled with organic matter oxidation
• Deep-sea SRB indeed performed DSR in deep-sea environment

## Graphical abstract

**Figure:**
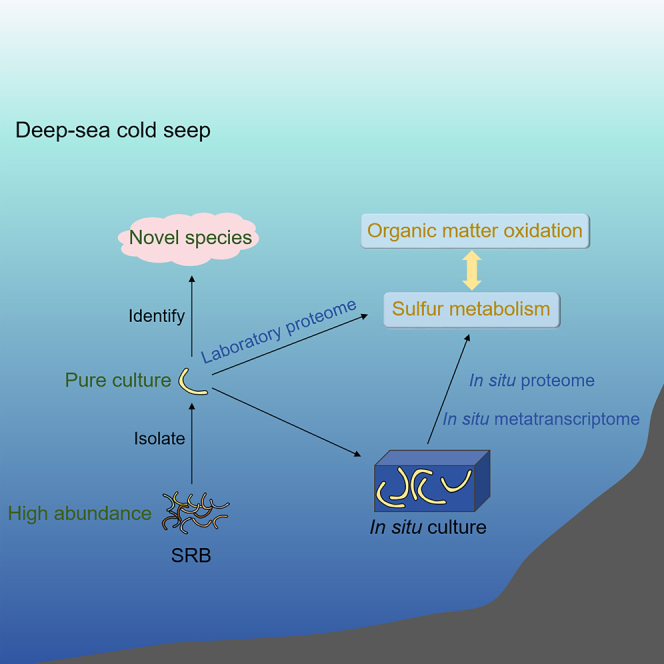

## Introduction

Sulfate-reducing bacteria are universally distributed in many engineered and natural environments where sulfate is present,^1^ which can obtain energy by oxidizing organic compounds or molecular hydrogen while reducing sulfate to hydrogen sulfide. Most SRB can also reduce other oxidized inorganic sulfur compounds, such as thiosulfate or polysulfide.^2^ It is known that SRB exist ubiquitously in deep oceans or terrestrial environments such as cold seeps, hydrothermal vents, oil reservoirs, aquifers, and groundwaters, where their geomicrobiological significance has often been emphasized.^3^^,^^4^ The growth of these organisms is highly favored by an anaerobic (oxygen-free) environment. SRB were one group of sulfate-reducing prokaryotes, and among the genera of SRB, the most thoroughly studied species are classified in the genus *Desulfovibrio*,^5^^,^^6^ which is the most common sulfate-reducing organism that can obtain its energy predominantly from the anaerobic reduction of sulfates. Sulfate reduction includes two approaches: assimilatory sulfate reduction and dissimilatory sulfate reduction. The dissimilatory sulfate reduction exists as a major reduction pathway in the species of the genus *Desulfovibrio*, *Pseudodesulfovibrio*, or *Oleidesulfovibrio* (a recently new genus made from *Desulfovibrio*), which involves the reduction of sulfate (SO_4_^2−^) to H_2_S (S^2−^). The reduction of sulfite (SO_3_^2−^) to H_2_S (S^2−^) mediated by dissimilatory sulfite reductase (DsrAB) is the most important part of the reduction process.^7^^,^^8^ As of now, more than 77 species of the genus *Desulfovibrio*, *Pseudodesulfovibrio*, or *Oleidesulfovibrio* have been described,^9^^,^^10^ which were isolated ubiquitously in nature, mainly from freshwater, and marine anaerobic systems.^11^^,^^12^ Many species of the genus *Desulfovibrio* have been isolated frequently from marine environments, including *Desulfovibrio senegalensis*,^9^ *Desulfovibrio frigidus*,^13^ *Desulfovibrio alkalitolerans*,^14^ *Desulfovibrio inopinatus*,^15^ and *Desulfovibrio bizertensis*.^16^ *Pseudodesulfovibrio* is a new genus originally proposed and reclassified of four species of the genus *Desulfovibrio* in 2016, and they all belong to the family *Desulfovibrionaceae*.^17^ Most species of the genus *Pseudodesulfovibrio* have been isolated from marine sediments, including *Pseudodesulfovibrio piezophilus*,^11^ *Pseudodesulfovibrio indicus*,^17^ *Pseudodesulfovibrio profundus*,^18^ *Pseudodesulfovibrio portus*,^4^ and *Pseudodesulfovibrio cashew*.^10^ The relatively high diversity and abundance of SRB have been reported in marine sediments, suggesting that SRB play a crucial role in the elemental cycles in marine sediments.^19^^,^^20^ For example, the sulfur cycle in marine sediments is primarily driven by the dissimilatory sulfate reduction to sulfide by the anaerobic SRB.^21^

Deep-sea cold seeps are widely distributed in the edges of continental shelves and mainly characterized by gas and liquid hydrocarbons from deep geological sources.^22^ The deep-sea cold seep is a very specific methane- and sulfate-rich environment, in which SRB account for approximately 5%–25% of microbial biomass in the surface of sulfate-rich zones and up to approximately 30%–35% in the sulfate-methane transition zone.^20^ The anaerobic oxidation of methane is the primary process of complex cold seep ecosystems, which is conjointly managed by a consortium of anaerobic methane-oxidizing archaea and SRB.^23^^,^^24^ However, only a few deep-sea SRB strains have been reported, limiting our understanding of their characteristics (e.g., material metabolism, element cycling, and ecological role). Thus, the deep-sea cold seep is one of the best locations to study deep-sea sulfur cycle mediated by SRB; it is utmost precious to obtain the typical SRB to explore their real metabolisms in this special environment.

In this study, we report the high abundance of SRB in deep-sea cold seep sediments. We isolated an anaerobic representative of deep-sea SRB from the deep-sea subsurface sediment. Combining physiological and proteomic approaches, we confirmed that strain zrk46 had strong ability to metabolize sulfate, thiosulfate, and sulfite via the dissimilatory sulfate reduction, accompanied by auxiliary metabolic effects of heterodisulfide reductase, ferredoxin, and nitrate reduction-associated proteins, in both laboratory and deep-sea *in situ* conditions. Lastly, the metatranscriptomic results also showed that deep-sea SRB indeed performed the sulfate reduction in deep-sea environment.

## Results and discussion

### Cultivation and morphology of a novel deep-sea sulfate-reducing bacterium

**Figure 1:**
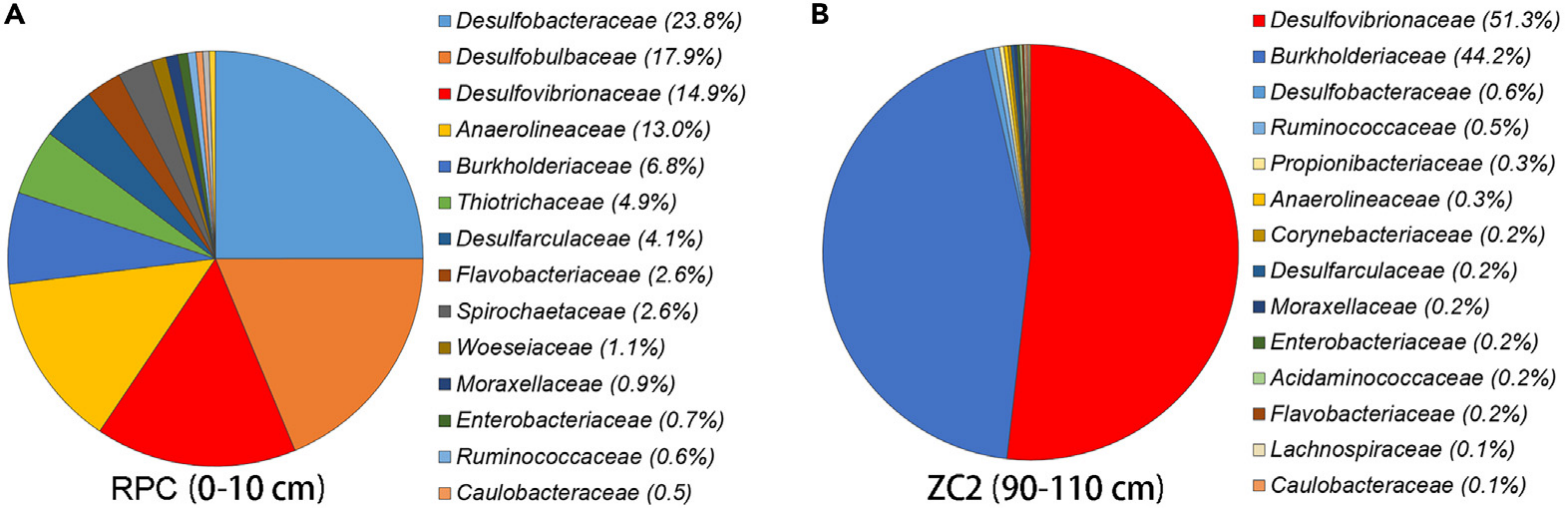
Detection of the abundance of SRB derived from deep-sea cold seep sediments The community structure of two sampling sites (A: RPC and B: ZC2) in the cold seep sediments as revealed by 16S rRNA gene amplicon profiling. The relative abundances of OTUs representing different bacteria are shown at the family level.

To gain preliminary insights of SRB existing in the deep-sea cold seep, operational taxonomic units sequencing was firstly performed to detect the relative abundance in SSU rRNA gene tag sequencing of SRB present in the deep-sea cold seep sediments. The result showed that *Desulfobacteraceae*, *Desulfobulbaceae*, and *Desulfovibrionaceae* were the top three families in surface sediment (RPC, 0–10 cm), all of which were sulfate-reducing bacterium (Figure 1A). The proportion of *Desulfovibrionaceae* accounted for 51.3% of the whole bacterial domain at the family level in sample ZC2 (90–110 cm) (Figure 1B), suggesting SRB were dominant in deep-sea cold seep sediments, which is similar to other marine environments.^20^ The diversity and abundance of SRB have been relatively high in deep-sea sediments, implying the vital importance of SRB in deep-sea environment.

**Figure 2:**
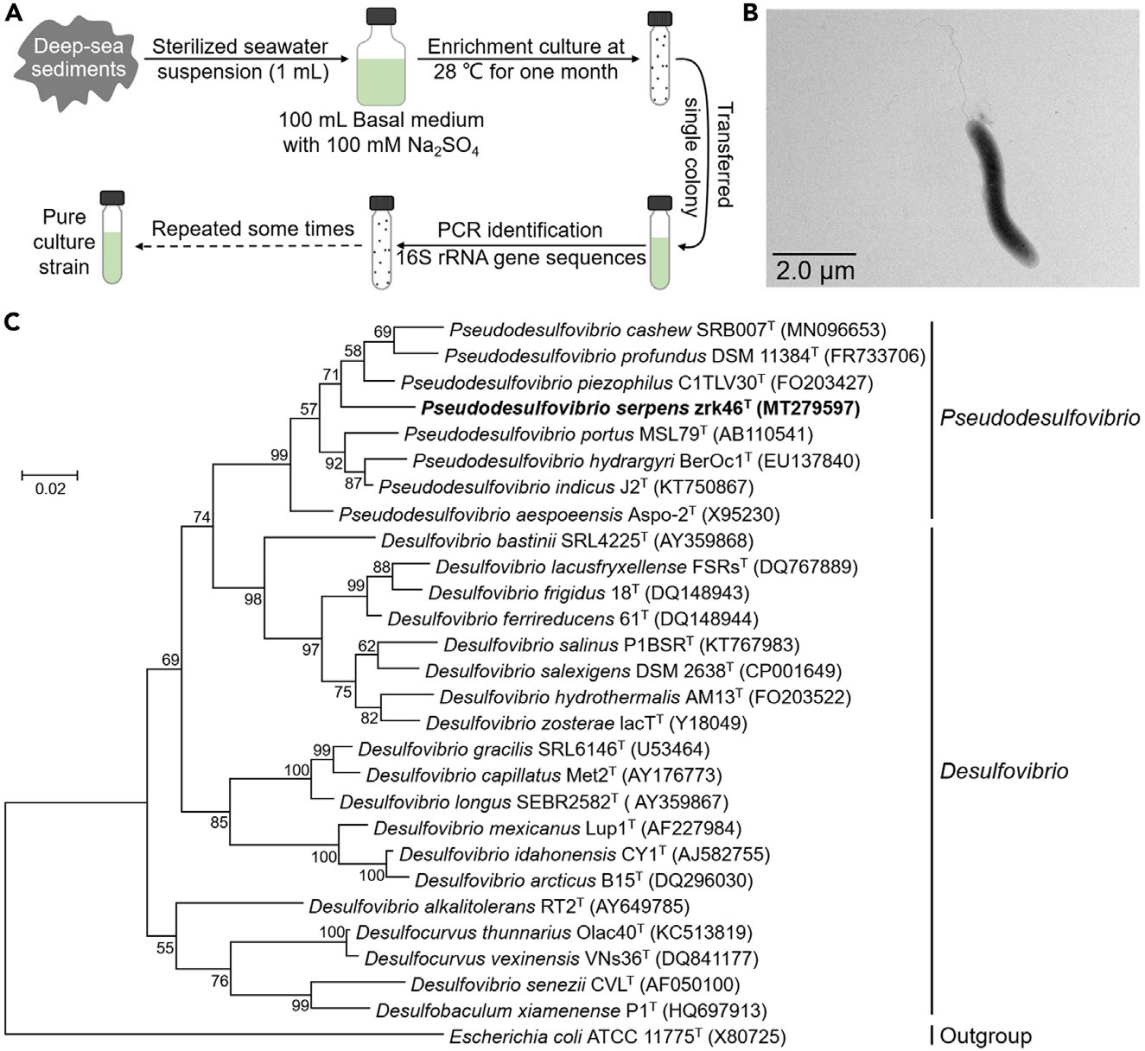
Isolation, morphology, and phylogenetic analyses of *Pseudodesulfovibrio serpens* zrk46 (A) Diagram showing the strategy used to isolate deep-sea SRB. (B) TEM observation of the morphology of strain zrk46. Scale bar, 2.0 μm. (C) Phylogenetic analysis of strain zrk46. Phylogenetic placement of strain zrk46 within the genus *Pseudodesulfovibrio*, based on almost complete 16S rRNA gene sequences. The NCBI accession number for each 16S rRNA gene is indicated after each corresponding strain’s name. The tree was inferred and reconstructed using the maximum likelihood criterion. Bootstrap values are based on 1,000 replicates. The 16S rRNA gene sequence of *Escherichia coli* ATCC 11775^T^ was used as the outgroup. Bar, 0.02 substitutions per nucleotide position.

To isolate SRB from deep-sea sediments, we developed an enrichment strategy by using a basal medium supplemented with 100 mM Na_2_SO_4_, which was often used as an electron acceptor for SRB. Then, these deep-sea sediment samples were anaerobically enriched at 28°C for one month. Thereafter, the enriched cultures were plated on the solid medium in Hungate tubes, and individual colonies with distinct morphology were picked and cultured (Figure 2A). Some of the cultured colonies were identified as SRB based on their 16S rRNA gene sequences. Among them, strain zrk46 possessed a fast growth rate and was chosen for further study. Under transmission electron microscopy observation, zrk46 showed a vibrioid or S-shaped, 2.0–4.5 × 0.3–0.7 μm in size, which was motile by means of a single polar flagellum (Figure 2B).

### Physiological characteristics, genome, and phylogeny of strain zrk46

The detailed physiological characteristics of strain zrk46 and the type strain *Pseudodesulfovibrio profundus* DSM 11384^T^ are listed in Table S1. Strain zrk46 grew at 0–80 g/L NaCl (optimum: 40 g/L) and grew at 16°C–45°C (optimum 28°C). The growth was observed at pH 6.0–8.5, the optimum being around 7.0. Compared with the type strain DSM 11384^T^, strain zrk46 showed its higher capacity to utilize different substrates as electron donors (fumarate, acetate, butyrate, formate, lactate, succinate, and malate) and acceptors (sulfate, sulfite, thiosulfate, and nitrate). The major fatty acids of strain zrk46 (>5% of the total) were iso-C_15:0_ (28.53%), anteiso-C_15:0_ (9.66%), C_16:1_*cis*9 (6.17%), C_16:0_ (17.29%), iso-C_17:0_ (6.21%), C_18:1_*cis*11/*trans*9/*trans*6 (6.88%), and C_18:0_ (7.42%) (Table S2). The amount of iso-C_15:0_, C_16:1_*cis*9, C_16:0_, iso-C_17:0_, C_18:1_*cis*11/*trans*9/*trans*6, and C_18:0_ in strain zrk46 was higher than that found in strain DSM 11384^T^. However, the amount of anteiso-C_15:0_ was lower in strain zrk46 (9.66%) than that found in strain DSM11384 (15.22%). The major polar lipids in strain zrk46 were phosphatidylethanolamine (PE), diphosphatidylglycerol (DPG), phosphatidylglycerol (PG), aminophospholipid (APL), and two phospholipids (PLs) (Figure S1).

To assess genomic features of strain zrk46, its whole genome was sequenced and analyzed (Figure S2). The chromosomal DNA G + C content of strain zrk46 was 53.26%, and its genome size was 4,082,342 bp. Strain zrk46 does not possess any plasmid. Annotation of the genome of strain zrk46 revealed it consisted of 3,717 predicted genes including 101 RNA genes (12 rRNA genes, 76 tRNA genes, and 13 other ncRNAs). To further clarify the phylogenetic position of strain zrk46, the genome relatedness values were calculated by the average nucleotide identity (ANI), *in silico* DNA-DNA similarity (*is*DDH), and the tetranucleotide signatures (Tetra), against the genomes of the four type strains of the genus *Pseudodesulfovibrio* (Table S3). The average nucleotide identities (ANIb) of strain zrk46 with strains Aspo-2^T^, J2^T^, C1TLV30^T^, and DSM 11384^T^ were 72.36%, 72.83%, 71.47%, and 73.31%, respectively. The average nucleotide identities (ANIm) of strain zrk46 with strains Aspo-2^T^, J2^T^, C1TLV30^T^, and DSM 11384^T^ were 83.43%, 83.63%, 83.85%, and 83.87%, respectively. The amino acid identities (AAI) of strain zrk46 with strains Aspo-2^T^, J2^T^, C1TLV30^T^, and DSM 11384^T^ were 74.5%, 73.9%, 76.4%, and 77.4%, respectively. The high AAI values to *Pseudodesulfovibrio* species identify strain zrk46 as a novel member of the genus *Pseudodesulfovibrio*. Based on digital DNA-DNA hybridization employing the Genome-to-Genome Distance Calculator GGDC,^25^ the *in silico* DDH estimates for zrk46 with strains Aspo-2^T^, J2^T^, C1TLV30^T^, and DSM 11384^T^ were 19.00%, 19.60%, 18.70%, and 19.90%, respectively. The tetranucleotide signatures (Tetra) of strain zrk46 with strains Aspo-2^T^, J2^T^, C1TLV30^T^, and DSM 11384^T^ were 0.75847, 0.73201, 0.75404, and 0.93565, respectively. These results demonstrated the genome of strain zrk46 to be clearly below established “cut-off” values (ANIb: 95%, ANIm: 95%, AAI: 95%, *is*DDH: 70%, TETRA: 0.99) for defining bacterial species,^26^ suggesting strain zrk46 represented a novel species within the genus *Pseudodesulfovibrio* as currently defined.

To further confirm the taxonomy of strain zrk46, we performed phylogenetic analyses. The maximum likelihood tree of 16S rRNA indicated that strain zrk46 belonged to the genus *Pseudodesulfovibrio* (Figure 2C). Based on the comparative 16S rRNA gene sequence analysis using BLAST tool and NCBI GenBank database, the 16S rRNA sequence similarity calculation using the NCBI server indicated that the closest relatives of strain zrk46 were *Pseudodesulfovibrio piezophilus* C1TLV30^T^ (96.11%), *Pseudodesulfovibrio aespoeensis* Aspo-2^T^ (95.27%), *Pseudodesulfovibrio profundus* DSM 11384^T^ (95.16%), and *Pseudodesulfovibrio indicus* J2^T^ (95.11%). Recently, the taxonomic threshold for species based on 16S rRNA gene sequence identity value was 98.65%.^27^ Together, based on phylogenetic, genomic, and phenotypic characteristics, we proposed that strain zrk46 might be a novel representative of the genus *Pseudodesulfovibrio*, for which the name *Pseudodesulfovibrio serpens* sp. nov. is proposed.

### Description of *Pseudodesulfovibrio serpens* sp. nov.

*Pseudodesulfovibrio serpens* (ser’pens. L. fem. n. serpens [italic type] the snake).

Cells are Gram-stain-negative, strictly anaerobic, S-shaped or vibrioid, 2.0–4.5 × 0.3–0.7 μm in size, motile by a single polar flagellum. Catalase positive, oxidase positive. Fumarate, acetate, butyrate, formate, lactate, succinate, and malate are oxidized with sulfate reduction. Sulfate, sulfite, thiosulfate, and nitrate could be used as electron acceptors. Growth is observed at salinities from 0 to 80 g/L NaCl (optimum: 40 g/L), from pH 6.0 to 8.5 (optimum 7.0) and at temperatures between 16°C and 45°C (optimum 28°C). The major polar lipids in strain zrk46 are PE, DPG, PG, APL, and two PLs. Major fatty acids (>10%) are iso-C_15:0_ (28.53%) and C_16:0_ (17.29%). The genome size of the type strain zrk46 is around 4.08 Mbp and the genomic DNA G + C content is 53.26%.

The type strain, zrk46 (=MCCC 1K04422^T^), is isolated from the cold seep in the South China Sea. The GenBank 16S rRNA gene sequence accession number for isolate zrk46 is MT279597 and the corresponding GenBank NCBI accession number of the genome sequence (CP051216).

### Effects of Na_2_SO_4_, Na_2_S_2_O_3_, Na_2_SO_3_, and Na_2_S on *P. serpens* zrk46 growth

**Figure 3:**
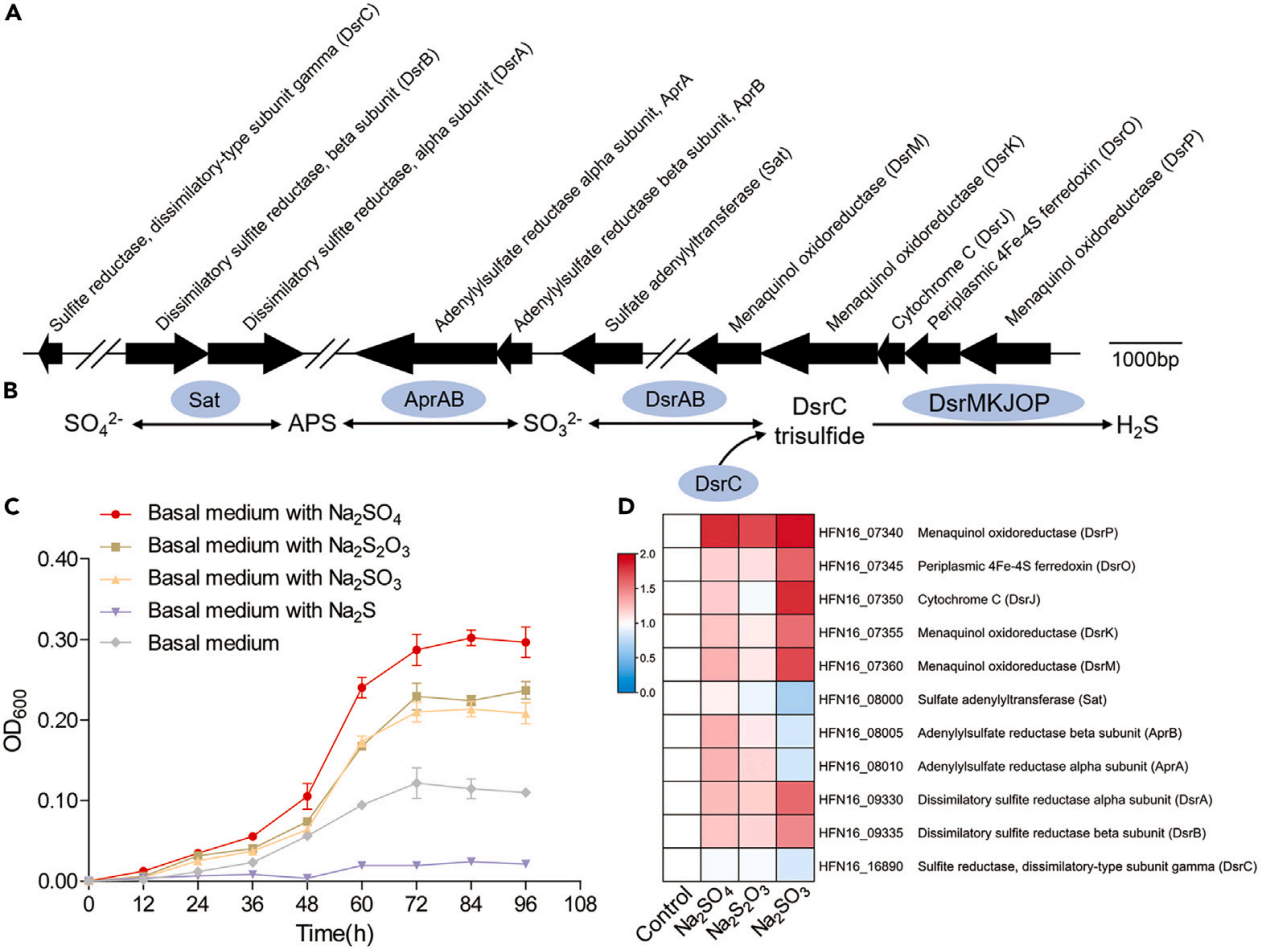
Sulfur metabolism assays of *Pseudodesulfovibrio serpens* zrk46 (A) The gene cluster containing the typical dissimilatory sulfate reductase operon and associated genes identified in the genome of strain zrk46. (B) Proposed pathway of dissimilatory sulfate reduction existing in strain zrk46. Sat, sulfur adenylyltransferase; APS, adenylyl sulfate; AprAB, adenylylsulfate reductase; DsrABC, reverse-type dissimilatory sulfite reductase; DsrMKJOP, sulfite reduction-associated complex. (C) Growth curves of strain zrk46 cultivated in basal medium alone and supplemented with either 20 mM Na_2_SO_4_, 20 mM Na_2_S_2_O_3_, 20 mM Na_2_SO_3_, or 5 mM Na_2_S. (D) Proteomics-based heatmap showing the relative expression levels of proteins associated with sulfur metabolism.

As SRB are essential anaerobic microorganisms mediating sulfur cycling,^28^ we firstly analyzed the sulfate reduction-associated genes in the strain zrk46 genome. As expected, the genes encoding dissimilatory sulfate reduction closely related proteins were present in the genome of strain zrk46 (Figure 3A). Inside the cytoplasm, sulfate is activated by the sulfate adenylyltransferase (Sat) to adenosine 5′-phosphosulfate (APS). Subsequently, APS is reduced to sulfite by the adenylylsulfate reductase (AprAB). Sulfite is a key intermediate in the dissimilatory sulfate reduction, which is reduced by the dissimilatory sulfite reductase (DsrAB) and a sulfur transfer protein (DsrC) together with the Dsr complex (DsrMKJOP) required for sulfate reduction (Figure 3B).^29^^,^^30^ To date, most studies on SRB have been related to lake,^31^ oil reservoirs,^32^ freshwater sediments,^33^ soil,^34^ gut,^35^ and so on, with only few reports on the deep-sea extreme environment. Here, we tested the effects of different sulfur-containing substances (including Na_2_SO_4_, Na_2_S_2_O_3_, Na_2_SO_3_, and Na_2_S) on strain zrk46 growth. These assays showed that adding Na_2_SO_4_, Na_2_S_2_O_3_, or Na_2_SO_3_ to the culture medium increased strain zrk46 growth (about 1.1–2 times), while adding Na_2_S inhibited growth (Figure 3C). Especially, the growth rate of strain zrk46 in the presence of Na_2_SO_4_ was about two times higher than that of control group (Figure 3C), indicating that strain zrk46 could effectively use sulfate (electron acceptor) and sodium lactate (electron donor) to perform the dissimilatory sulfate reduction. To better understand the dissimilatory sulfate reduction of strain zrk46, we performed proteomic analysis of strain zrk46 cultured in basal medium alone or supplemented with either Na_2_SO_4_, Na_2_S_2_O_3_, or Na_2_SO_3_ to explore the underlying mechanism of growth promotion. We found that most dissimilatory sulfate reduction-associated proteins were simultaneously upregulated in the presence of Na_2_SO_4_, Na_2_S_2_O_3_, and Na_2_SO_3_ (Figure 3D). These results showed that strain zrk46 could effectively reduce sulfate, thiosulfate, and sulfite to hydrogen sulfide via dissimilatory sulfate reduction, from which it can obtain energy to promote its growth.

**Figure 4:**
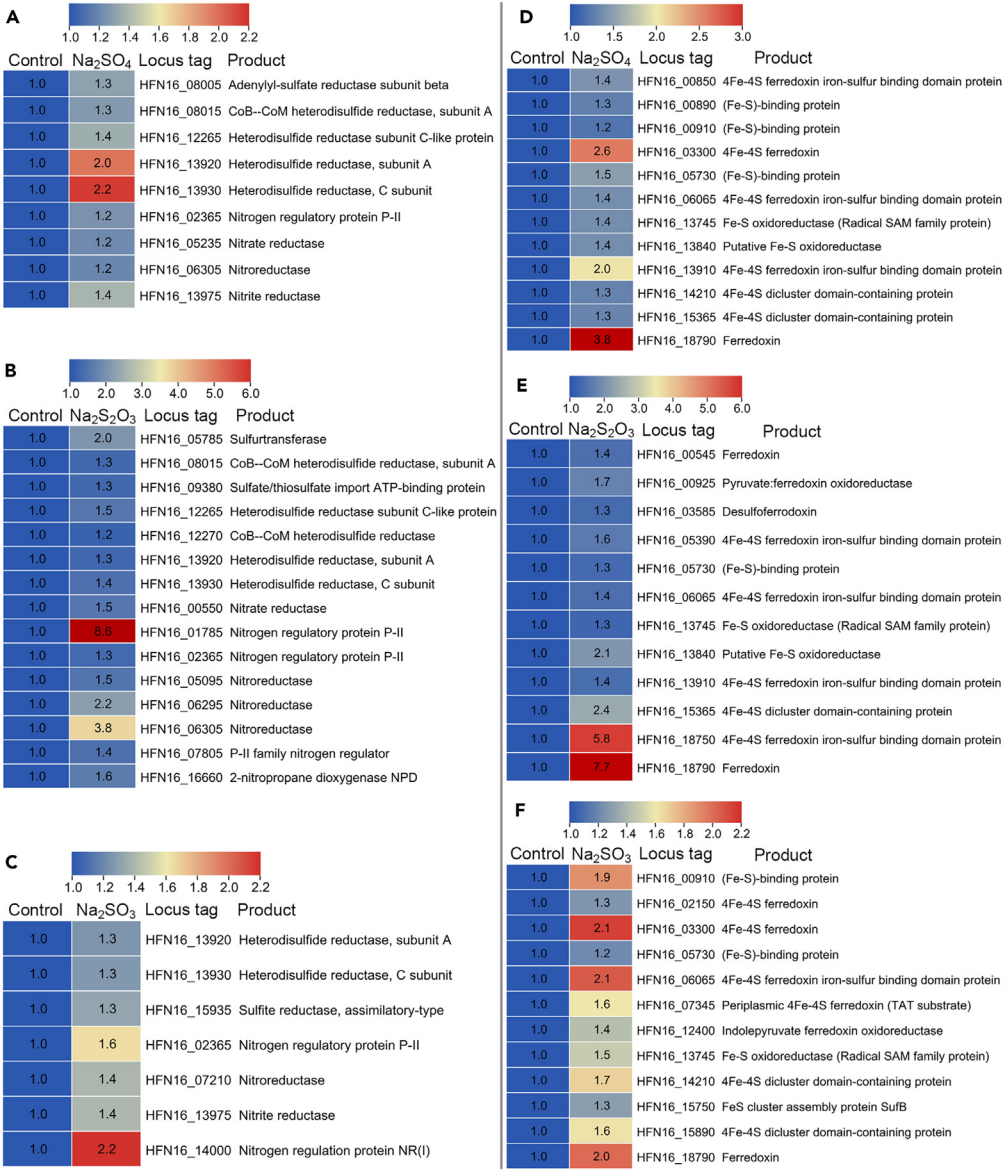
Proteomic analysis of *Pseudodesulfovibrio serpens* zrk46 cultivated in basal medium alone and supplemented with either 20 mM Na_2_SO_4_, 20 mM Na_2_S_2_O_3_, or 20 mM Na_2_SO_3_ Proteomics-based heatmap showing the upregulated proteins associated with sulfur metabolism and nitrogen metabolism (A, B, and C). Proteomics-based heatmap showing the upregulated Fe-S-associated proteins (D, E, and F). “Control” indicates basal medium. “Na_2_SO_4_, Na_2_S_2_O_3_, and Na_2_SO_3_” indicate basal medium supplemented with 20 mM Na_2_SO_4_, 20 mM Na_2_S_2_O_3_, and 20 mM Na_2_SO_3_, respectively.

Moreover, we observed that the expression of heterodisulfide reductase-related proteins was simultaneously upregulated in the presence of Na_2_SO_4_, Na_2_S_2_O_3_, and Na_2_SO_3_ (Figures 4A–4C). Heterodisulfide reductase was widespread in the domains Bacteria and Archaea and play roles in a greater diversity of energy-conserving metabolisms, including the reduction of sulfate^36^^,^^37^ and the oxidation of inorganic sulfur compounds.^38^ Therefore, the result indicated that heterodisulfide reductase could assist the sulfate reduction process of strain zrk46. Interestingly, the expression of nitrogen metabolism-associated proteins (including nitrate reductase, nitrite reductase, and nitroreductase) was simultaneously upregulated in the presence of Na_2_SO_4_, Na_2_S_2_O_3_, and Na_2_SO_3_ (Figures 4A–4C), indicating that these reductases might play a role in the sulfate reduction process of strain zrk46, which has never been previously reported and needs further studies. Although they cannot directly affect inorganic sulfur compounds, they might contribute significantly to the electron transfer and formation energy of strain zrk46. Alternatively, the expression of iron-sulfur (Fe-S) proteins and ferredoxins was also upregulated in the presence of Na_2_SO_4_, Na_2_S_2_O_3_, and Na_2_SO_3_ (Figures 4E–4F). Ferredoxins comprise a large family of Fe-S proteins that transfer electrons in diverse biological processes.^39^ The type Fe-S proteins in strain zrk46 was 2Fe-2S cluster and 4Fe-4S cluster, but only the expressions of 4Fe-4S-associated proteins were upregulated, indicating that it played a functional role in electron transfer processes relevant for the sulfate reduction process of strain zrk46. In the future, the genetic operating system of this strict anaerobic bacterium needs to be constructed to further confirm its function.

### Proteomic analysis of *P. serpens* zrk46 cultured in deep-sea conditions

**Figure 5:**
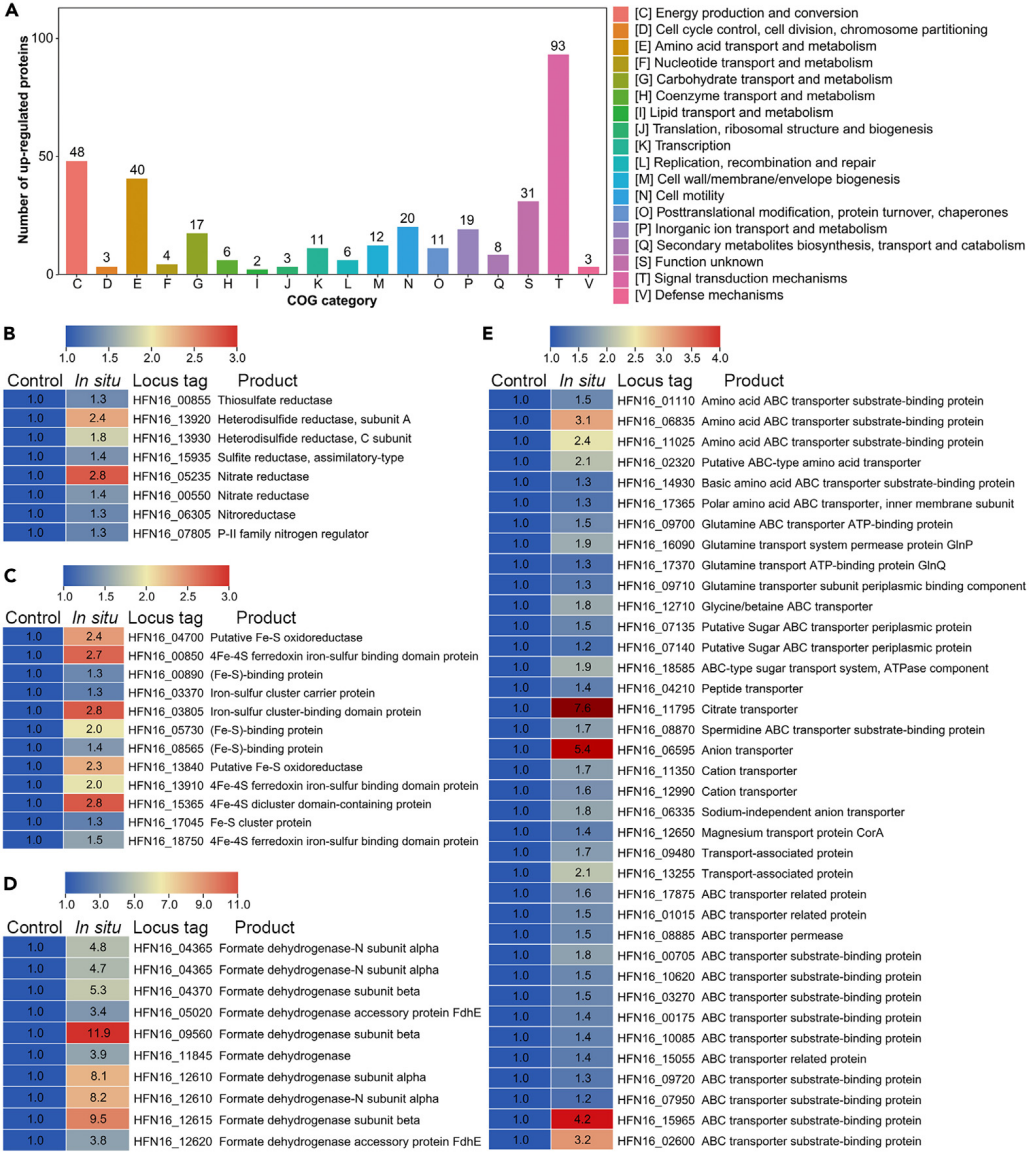
Proteomic analysis of *Pseudodesulfovibrio serpens* zrk46 incubated in the deep-sea cold seep (A–E) COG category showing the significant enrichment of upregulated proteins in strain zrk46 cultivated in the deep-sea cold seep. Proteomics-based heatmap showing the upregulated proteins associated with sulfur metabolism and nitrogen metabolism (B), Fe-S proteins (C), formate dehydrogenase (D), and ABC transporter proteins (E). “Control” indicates strain zrk46 cultured in deep-sea condition without exchanging with outside; “*In situ*” strain zrk46 cultured in deep-sea condition exchanging with outside.

Considering stain zrk46 was isolated from the deep-sea environment, we next sought to explore its actual metabolisms performed in the deep-sea environment. We thus performed the *in situ* cultivation of strain zrk46 in anaerobic bags either without or with exposure to the deep-sea environment (where we isolated this bacterium) for 10 days, as previously described.^40^ Subsequently, the strain zrk46 cells were collected, and proteomic analyses were performed. The upregulated proteins were predominantly mapped to COG categories representing energy production and conversion, amino acid transport and metabolism, cell motility, and signal transduction mechanisms (Figure 5A). Interestingly, the proteomic results clearly showed that the expressions of thiosulfate reductase, heterodisulfide reductase, and sulfite reductase were upregulated when compared to that cultured under the closed condition (Figure 5B), which were consistent with the laboratory conditions, indicating dissimilatory sulfate reduction indeed happened in the deep sea. Through the analysis of genomic and proteomic data under different substrates and conditions, we found that *sat*, *dsrA*, *dsrB*, *dsrP*, *dsrM*, *dsrK*, *dsrO*, *hdrA*, and *hdrC* might be essential genes and proteins expressed in the sulfur metabolism of strain zrk46.^8^ The expressions of nitrate reductase and nitroreductase were also upregulated and the nitrate concentration was 24.3 μM in the *in situ* seawater,^41^ which indicated that strain zrk46 performed active nitrogen metabolism in the deep sea, or it could assist strain zrk46 to acquire electrons from electron transfer chains to reduce sulfate, which awaits future studies.^42^ Consistently, the expressions of most 4Fe-4S proteins and ferredoxins were upregulated in the deep sea (Figure 5C), indicating that they indeed play a vital role in the deep-sea environment, possibly involved in electron transfer during the sulfate reduction of strain zrk46. In addition, the formate dehydrogenases were also highly expressed in the deep-sea environment (Figure 5D), suggesting that strain zrk46 could enhance formate metabolism and thus providing more electrons and protons (reducing power) for the sulfate reduction process.^43^ In anoxic environments, SRB are primarily responsible for organic carbon oxidation, because sulfate is often the predominant electron acceptor.^28^^,^^44^ It has previously been reported that sulfate reduction could facilitate the organic matter oxidation up to 50% in the marine sediments.^45^ Notably, most ABC transporters (associated with amino acids, sugars, ions etc.) were upregulated (Figure 5E), indicating that strain zrk46 could effectively ingest and degrade the organic compounds coupled with sulfate reduction process in the deep-sea environment.^46^^,^^47^ Therefore, these results showed that deep-sea SRB might contribute to the oxidation of organic matter in the deep sea by dissimilatory sulfate reduction, which could allow them to metabolically thrive in extreme habitats. In conclusion, we performed the *in situ* experiments on deep-sea sulfate-reducing bacterium zrk46 to reveal its true metabolic characteristics in deep-sea environment, strongly suggesting that SRB may play undocumented roles in deep-sea sulfur and carbon cycling.

### *In situ* metatranscriptomic analysis of the metabolism of deep-sea SRB

**Figure 6:**
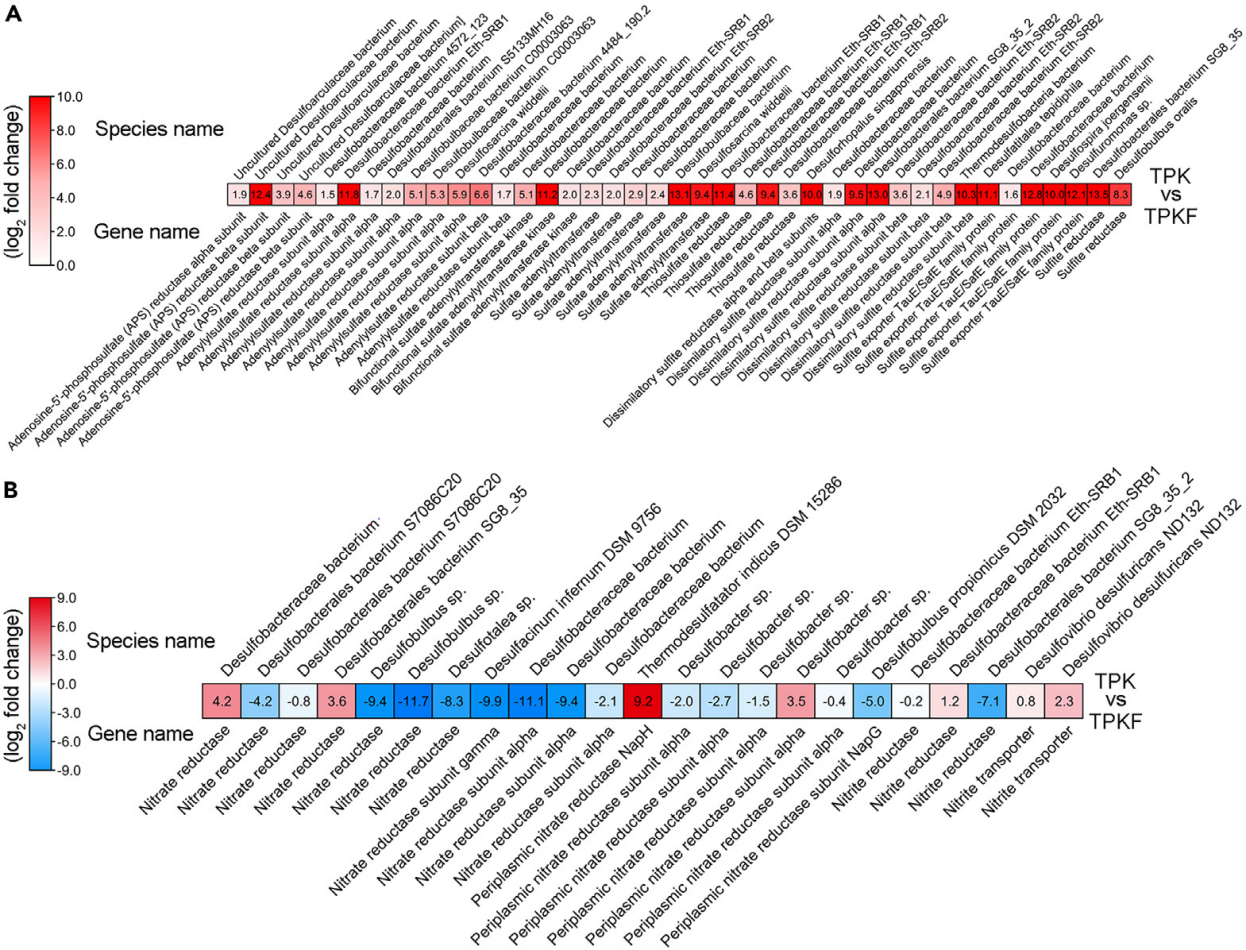
*In situ* metatranscriptomic analysis of the metabolic characteristics of deep-sea SRB (A and B) The relative expression levels of genes encoding proteins associated with sulfur metabolism (A) and nitrogen metabolism (B) in deep-sea SRB. The numbers in A and B represent the fold change of gene expression (by using the log_2_ value). TPK, sedimental sample from the cold seep vent; TPKF, sedimental sample far away from the cold seep vent.

Given the high abundance of SRB and the real sulfur metabolism of strain zrk46 in the deep-sea cold seep, we performed the metatranscriptomic sequencing analysis to investigate real metabolisms of other deep-sea SRB. The result showed that the genes encoding APS reductase, adenylylsulfate reductase, sulfate adenylyltransferase, thiosulfate reductase, dissimilatory sulfite reductase, and sulfite exporter of different SRB were upregulated in the cold seep sediment (where strain zrk46 was isolated) compared to the sediment that was far away from the cold seep (Figure 6A). These genes are all important for the sulfate reduction, indicating that SRB indeed perform the sulfate reduction in deep-sea environment and play a pivotal role in the sulfur biogeochemical cycling.^48^ Consistent with our separation strategy, in the future, we can enrich and culture deep-sea SRB based on a sulfate-driven sulfate reduction process. In contrast, the genes encoding nitrate reductase and nitrite reductase of most SRB were downregulated, while only a few were upregulated (Figure 6B), possibly due to the high concentration of sulfate and low concentration of nitrate in the deep-sea cold seep.^41^ These upregulated nitrate reductases or nitrite reductases in SRB might assist them to acquire electrons from electron transfer chains in the sulfate reduction, which awaits future studies.

### Conclusion

The integrative analysis of genomic, physiological, laboratory and *in situ* proteomic, and metatranscriptomic data in the present study identified the sulfur metabolic characteristics of a novel deep-sea sulfate-reducing bacterium (zrk46). Our findings indicate that the deep-sea SRB can perform dissimilatory sulfate reduction in both laboratory and deep-sea conditions, which expands our knowledge of the sulfur metabolic potential of deep-sea SRB and provides hints to roles for deep-sea SRB in the marine sedimentary sulfur and carbon cycle. Moreover, we reveal that diverse genes related to sulfur metabolism in deep-sea SRB were upregulated, suggesting that deep-sea SRB might play a pivotal role in the deep-sea sulfur biogeochemical cycling.

## STAR★Methods

### Key resources table

**Table:** 

REAGENT or RESOURCE	SOURCE	IDENTIFIER
**Bacterial and virus strains**		

Strain zrk46	This paper	N/A
*Pseudodesulfovibrio profundus* DSM 11384	Marine Culture Collection of China, MCCC	N/A

**Chemicals, peptides, and recombinant proteins**		

Sodium lactate	Sinopharm	CAS：72-17-3
Sodium sulfate	Sinopharm	CAS：7757-82-6
Sodium sulfite	Sinopharm	CAS：7757-83-7
Sodium thiosulfate	Sinopharm	CAS：7772-98-7
Sodium sulfide	Sinopharm	CAS：1313-82-2
Sodium nitrate	Sinopharm	CAS：7631-99-4
Sodium nitrite	Sinopharm	CAS：7632-00-0
Ammonium chloride	Sinopharm	CAS：12125-02-9
Sodium bicarbonate	Sinopharm	CAS：144-55-8
Sodium acetate	Sinopharm	CAS：127-09-3
Potassium dihydrogen phosphate	Sinopharm	CAS：7778-77-0
Magnesium sulfate	Sinopharm	CAS：10034-99-8
Cysteine hydrochloride	solarbio	CAS：52-89-1
Sodium resazurin	solarbio	CAS：62758-13-8
PBS buffer	solarbio	N/A

**Deposited data**		

Amplicon sequencing	NCBI Short Read Archive	Short Read Archive: PRJNA675395
16S rRNA gene sequence	NCBI GenBank	GenBank: MT279597
Nanopore DNA genome sequence	NCBI GenBank	GenBank: CP051216
Proteomics	ProteomeXchange	ProteomeXchange: PXD043318
Metatranscriptomics	NCBI Short Read Archive	Short Read Archive: PRJNA988008

**Software and algorithms**		

GraphPad Prism 8.2.0	GraphPad Software	https://www.graphpad.com/scientificsoftware/prism/

### Resource availability

#### Lead contact

Further information and requests for resources and reagents should be directed to and will be fulfilled by the lead contact Chong Wang (wangchongyilin@163.com).

#### Materials availability

This study did not generate new unique materials or reagents.

#### Data and code availability

• Data: All data reported in this paper will be shared by the lead contact upon request.
• Code: This paper does not report original code.
• Any information required to analyze the data reported in this paper is available upon request from the lead contact.

### Experimental model and study participant details

All experiments in this study were performed with *Pseudodesulfovibrio* strain zrk46 and the type strain-*Pseudodesulfovibrio profundus* DSM 11384^T^. Strain zrk46 was isolated from the deep-sea cold seep sediment samples (E 119°17′07.322″, *N* 22°06′58.598''; at a depth of approximately 1,146 m) in this study. *Pseudodesulfovibrio profundus* DSM 11384 was obtained from the Marine Culture Collection of China, MCCC.

### Method details

#### Sampling and OTUs analysis

The deep-sea sediment samples (temperature, 3.64°C; pH, 7.62; salinity, 3.449%) were collected by *RV KEXUE* from a typical cold seep in the South China Sea (E 119°17′07.322″, *N* 22°06′58.598'') at a depth of approximately 1,146 m in July of 2018. We selected two sedimentary samples (RPC and ZC2 at depth intervals of 0–10 and 90–110 cm, respectively) for OTUs sequencing that performed by Novogene (Tianjin, China). Briefly, total DNAs from these samples were extracted by a CTAB/SDS method^49^ and diluted to 1 ng/μL with sterile water, and then used for PCR template. We used the primers (341F: 5′- CCTAYGGGRBGCASCAG and 806R: 5′- GGACTACNNGGGTATCTAAT) to amplify the 16S rRNA genes of distinct regions (V3/V4). Then, we used a Qiagen Gel Extraction Kit (Qiagen, Germany) to purify these PCR products for libraries construction. Sequencing libraries were generated using TruSeq DNA PCR-Free Sample Preparation Kit (Illumina, USA) following the manufacturer’s instructions, and then assessed on the Qubit@ 2.0 Fluorometer (Thermo Scientific, USA) and Agilent Bioanalyzer 2100 system. The library was sequenced on an Illumina NovaSeq platform and 250 bp paired-end reads were generated and merged using FLASH (V1.2.7, http://ccb.jhu.edu/software/FLASH/).^50^ Quality filtering on raw tags was performed according to the QIIME (V1.9.1, http://qiime.org/scripts/split_libraries_fastq.html) quality controlled process to obtain the high-quality clean tags.^51^ To detect chimera sequences,^52^ the tags were compared with the reference database (Silva database version 138, https://www.arb-silva.de/) using UCHIME algorithm (UCHIME Algorithm, http://www.drive5.com/usearch/manual/uchime_algo.html).^53^ Sequence analyses were performed by Uparse software (Uparse v7.0.1001, http://drive5.com/uparse/)^54^ and the sequences with ≥97% similarity were assigned to the same OTUs. The representative sequence for each OTU was screened for further annotation. Finally, we used the Silva Database (http://www.arb-silva.de/)^55^ with the Mothur algorithm to annotate taxonomic information.

#### Enrichment and cultivation of deep-sea SRB

To isolate and cultivate deep-sea SRB, 1.0 g deep-sea sediment samples of RPC or ZC2 was suspended with 1 mL sterilized seawater, respectively, and then added to a 250 mL anaerobic bottle containing 100 mL basal medium (containing 2.0 g/L sodium lactate, 1.0 g/L NH_4_Cl, 1.0 g/L NaHCO_3_, 1.0 g/L CH_3_COONa, 0.5 g/L KH_2_PO_4_, 0.2 g/L MgSO_4_^**.**^7H_2_O, 0.7 g/L cysteine hydrochloride, 500 μL/L 0.1% (w/v) resazurin in 1 L seawater, pH 7.0) supplemented with 100 mM Na_2_SO_4_ under a 100% N_2_ atmosphere. The medium was prepared under a 100% N_2_ gas phase and sterilized by autoclaving at 115°C for 30 min. The inoculated media were anaerobically incubated at either 4°C or 28°C for one month. The basal medium supplemented with 100 mM Na_2_SO_4_ and 15 g/L agar was evenly spread on to the inside wall of a Hungate tube, which formed a thin layer of medium for the bacteria to grow. After this, 100 μL of the enriched culture was anaerobically transferred into the anaerobic roll tubes and then spread on to the medium layer. These tubes were also anaerobically cultured at 28°C for three days. Single colonies were selected using sterilized bamboo sticks; they were then cultured in the 15 mL Hungate tube containing 10 mL basal medium supplemented with 100 mM Na_2_SO_4_ at 28°C for three days under a 100% N_2_ atmosphere. Thereafter, the amplification and sequencing of 16S rRNA genes were performed to identify these cultures. The primers 27F (5′-AGAGTTTGATCCTGGCTCAG-3′) and 1492R (5′- GGTTACCTTGTTACGACTT-3′) were used to amplify 16S rRNA gene sequences. PCR conditions were set as following: pre-denaturation at 95 °C for 10 min; denaturation at 95 °C for 15 s, annealing at 54 °C for 15 s, extension at 72 °C for 20 s, in 30 cycles; and final extension at 72 °C for 5 min. These PCR amplification products were sequenced in Tsingke Biotechnology Co., Ltd (Beijing, China), and the sequences were analyzed by BLASTn of the NCBI database. One strain (zrk46) was identified as a member of the genus *Pseudodesulfovibrio*, but was noted to have less than 97% 16S rRNA gene sequence similarity to other cultured strains; strain zrk46 was therefore selected and purified by repeating the Hungate roll-tube method. The purity of strain zrk46 was confirmed regularly by repeating partial sequencing of the 16S rRNA gene and by observation using a transmission electron microscope (TEM).

#### TEM observation

To observe the morphological characteristics of strain zrk46, 10 mL culture was collected by centrifuging at 4000 × *g* for 5 min. Cells were then washed three times with PBS buffer (137 mM NaCl, 2.7 mM KCl, 10 mM Na_2_HPO_4_, 1.8 mM KH_2_PO_4_, 1 L sterile water, pH 7.4). Finally, the cells were suspended in 20 μL PBS buffer, and then transferred onto copper grids coated with a carbon film by immersing the grids in the cell suspension for 30 min.^56^ All samples were examined under TEM (HT7700, Hitachi, Japan).

#### Genome sequencing and analysis

Genomic DNA of strain zrk46 was extracted from 0.5 L cells that cultured for three days at 28°C. The DNA library was prepared using the Ligation Sequencing Kit (SQK-LSK109, UK), and sequenced using an FLO-MIN106 vR9.4 flow-cell for 48 h on MinKNOWN software v1.4.2 (Oxford Nanopore Technologies, UK) at the Beijing Novogene Bioinformatics Technology Co., Ltd. The detailed sequencing procedures were performed as previously described.^40^ The experimental process was implemented in accordance with the standard protocol provided by Oxford Nanopore Technologies (ONT), including sample quality testing, library construction, library quality testing and library sequencing. Library construction includes the following steps: (i) High quality genomic DNA was extracted, and then Nanodrop, Qubit and 0.35% agarose gel electrophoresis were used for purity, concentration and integrity inspection; (ii) The large fragments of DNA were recovered by the BluePippin automatic nucleic acid recovery system; (iii) Library construction was performed with the SQK-LSK109 connection kit, and the construction procedures included DNA damage repair and terminal repair, magnetic bead purification, ligation of sequencing adapters and magnetic bead purification. After library construction, computer sequencing was performed. Canu V1.5 software^57^ was used to assemble the filtered subreads. Finally, Pilon software^58^ was used to further correct the assembled genome with second-generation data to obtain the final genome with higher accuracy. Moreover, the genome relatedness values were calculated by several approaches: amino acid identity (AAI), average nucleotide identity (ANI) based on the MUMMER ultra-rapid aligning tool (ANIm), ANI based on the BLASTN algorithm (ANIb), the tetranucleotide signatures (TETRA), and *in silico* DNA–DNA similarity (*is*DDH). The amino acid identity (AAI) values were calculated by AAI-profiler (http://ekhidna2.biocenter.helsinki.fi/AAI/).^59^ ANIm, ANIb, and TETRA frequencies were calculated by the JSpecies WS (http://jspecies.ribohost.com/jspeciesws/).^60^ The *is*DDH values were calculated by the Genome-to-Genome Distance Calculator (GGDC) (http://ggdc.dsmz.de/).^25^ The *is*DDH results were based on the recommended formula 2, which is independent of genome size. The recommended species criterion cut-offs were used: 95% for the AAI, ANIb and ANIm, 0.99 for the TETRA signature, and 70% for the *is*DDH.^26^

#### Phenotypic characteristics analyses

Unless stated otherwise, physiological characterization was carried out anaerobically in basal medium supplemented with 1.0 g/L yeast extract, 1.0 g/L peptone, and 20 mM Na_2_SO_4_. Growth was tested at different temperatures (4, 16, 28, 30, 32, 37, 45, 60, 70, 80°C) for 10 days. The pH range for growth was tested from pH 4.0 to pH 10.0 with increments of 0.5 pH units. The pH of the culture medium was adjusted by 6 M HCl for low pH and 10% NaHCO_3_ (w/v) for high pH. Salt tolerance was tested on the modified basal medium (replaced sea water with distilled water) supplemented with 0–10.0% (w/v) NaCl (1% intervals) for ten days. Determination of electron donors and acceptors for growth were performed with different electron donors at 20 mM (acetate, fumarate, formate, pyruvate, lactate, malate, methanol, fructose, propionate, butyrate, succinate, glycine, ethanol) and different electron acceptors: elemental sulfur (1%, w/v), sulfate (20 mM), sulfite (20 mM), thiosulfate (20 mM), nitrate (20 mM), and nitrite (20 mM). For each substrate, three biological replicates were performed.

For chemotaxonomic analysis, cells of zrk46 were cultured and collected under the same conditions unless stated otherwise, with the closely related type strain (*Pseudodesulfovibrio profundus* DSM 11384^T^) were grown on basal solid medium for 4 days at 28°C under the same condition. Cells of zrk46 and the closely related type strain were harvested from cultures at the mid-exponential phase of growth and freeze-dried. Cellular fatty acids were extracted and determined from dried cells by using GC (model 7890A, Agilent, USA) according to the protocol of the Sherlock Microbial Identification System. Polar lipids were extracted and determined as described by Tindall et al.*.*^61^

#### Phylogenetic analysis

To construct a maximum likelihood 16S rRNA phylogenetic tree, the full-length 16S rRNA gene sequences of strain zrk46 and other related taxa were obtained from the NCBI database (www.ncbi.nlm.nih.gov/). The phylogenetic tree was constructed using the W-IQ-TREE web server (http://iqtree.cibiv.univie.ac.at)^62^ with the “GTR+F+I + G4” model, and the Interactive Tree of Life (iTOL v5) online tool^63^ was used to edit the phylogenetic trees.

#### Growth assays of strain zrk46

To assess the effects of different inorganic sulfur sources (20 mM Na_2_SO_4_, 20 mM Na_2_S_2_O_3_, 20 mM Na_2_SO_3_, and 5 mM Na_2_S) on strain zrk46 growth, we used a basal culture medium supplemented with the sulfur sources mentioned above. For each growth assay, 1 mL of strain zrk46 culture was inoculated in a 250 mL Hungate bottle containing 100 mL of the respective media. All Hungate bottles were anaerobically incubated at 28 °C. Bacterial growth was monitored by measuring OD_600_ values every 12 h via a microplate reader until cell growth reached a stationary phase. Three replicates were performed for each condition.

#### Proteomic analysis of sulfur metabolism of strain zrk46 cultured in laboratory condition

For proteomic analysis, cells suspension of strain zrk46 cultured in 200 mL of either basal medium or basal medium supplemented with different sulfur compounds (20 mM Na_2_SO_4_, 20 mM Na_2_S_2_O_3_, or 20 mM Na_2_SO_3_) at 28 °C for four days were collected at 8,000 ×*g* for 20 min. Subsequently, the cells were collected and sonicated three times on ice using a high intensity ultrasonic processor in lysis buffer (8 M urea, 1% Protease Inhibitor Cocktail). The remaining debris was removed by centrifugation at 12,000 ×*g*, 4°C for 10 min. Finally, the supernatant was collected and the protein concentration was determined with a BCA kit (Solarbio, China) according to the instructions. Proteomic sequencing analysis was performed by PTMBiolabs (Hangzhou, China), and the detailed protocols of proteomic sequencing were shown below.

##### Sample processing protocol

For trypsin digestion, the protein solution was reduced with 5 mM dithiothreitol for 30 min at 56°C and alkylated with 11 mM iodoacetamide for 15 min at room temperature in darkness. The 100 mm TEAB was added to the diluted protein sample in a solution with a urea concentration of less than 2 M. Finally, trypsin was added at a trypsin to protein mass ratio of 1:50 for the first digestion overnight, with 1:100 trypsin and protein. The mass was added for a second digestion for 4 h. Then the tryptic peptides were dissolved in 0.1% formic acid (solvent A) and directly loaded into a home-made reversed-phase analytical column (15-cm length, 75 μm inner diameter). The gradient increased from 6% to 23% in solvent B (0.1% formic acid in 98% acetonitrile) over 26 min, from 23% to 35% in 8 min and increased to 80% in 3 min, then maintain 80% for the last 3 min, and all at a constant flow rate of 400 nL/min on an EASY-nLC 1000 UPLC system.

The peptides were coupled to UPLC in Q ExactiveTM Plus (Thermo, USA) via NSI source and tandem mass spectrometry (MS/MS). The applied electrospray voltage was 2.0 kV. The full scan has an m/z scan range of 350 to 1,800, and at 70,000 resolution, intact peptides were detected in the Orbitrap. MS/MS was then selected using the NCE set to 28 select peptides and fragments were detected in the Orbitrap at a resolution of 17,500. A data-related process that alternates between one MS scan followed by 20 MS/MS scans with 15.0 s dynamic exclusion. The automatic gain control (AGC) was set to 5E4. The fixed first mass was set as 100 m/z.

##### Data processing protocol

(1) Database Search. The resulting MS/MS data were processed using Maxquant search engine (v.1.5.2.8).^64^ Tandem mass spectra were searched against some databases (such as UniProt-GOA, InterPro, Kyoto Encyclopedia of Genes and Genomes (KEGG)) concatenated with reverse decoy database. Trypsin/P was specified as cleavage enzyme allowing up to 2 missing cleavages. The mass tolerance for precursor ions was set as 20 ppm in First search and 5 ppm in Main search, and the mass tolerance for fragment ions was set as 0.02 Da. Carbamidomethyl on Cys was specified as fixed modification and oxidation on Met was specified as variable modifications. FDR was adjusted to <1% and minimum score for peptides was set >40. (2) Enrichment of Gene Ontology analysis. Proteins were classified by GO annotation into three categories: biological process, cellular compartment and molecular function. For each category, a two-tailed Fisher’s exact test was employed to test the enrichment of the differentially expressed protein against all identified proteins. The GO with a corrected *p*-value <0.05 is considered significant. (3) Enrichment of pathway analysis. Encyclopedia of Genes and Genomes (KEGG) database was used to identify enriched pathways by a two-tailed Fisher’s exact test to test the enrichment of the differentially expressed protein against all identified proteins.^65^ The pathway with a corrected *p*-value <0.05 was considered significant. These pathways were classified into hierarchical categories according to the KEGG website. (4) Enrichment of protein domain analysis. For each category proteins, InterPro (a resource that provides functional analysis of protein sequences by classifying them into families and predicting the presence of domains and important sites) database was researched and a two-tailed Fisher’s exact test was employed to test the enrichment of the differentially expressed protein against all identified proteins. Protein domains with a *p*-value <0.05 were considered significant. (5) Enrichment-based Clustering. For further hierarchical clustering based on different protein functional classification (such as: GO, Domain, Pathway, Complex). We first collated all the categories obtained after enrichment along with their *p* values, and then filtered for those categories which were at least enriched in one of the clusters with *p* value < 0.05. This filtered *p* value matrix was transformed by the function x = −log_10_ (*p* value). Finally these x values were z-transformed for each functional category. These z scores were then clustered by one-way hierarchical clustering (Euclidean distance, average linkage clustering) in Genesis. Cluster membership was visualized by a heatmap using the “heatmap.2” function from the “gplots” R-package.

#### Proteomic analysis of sulfur metabolism of strain zrk46 cultured in the deep sea

To explore the actual metabolic characteristics of strain zrk46 conducted in the deep-sea cold seep, the *in situ* cultivation was performed. Briefly, 2 mL freshly incubated strain zrk46 cells was respectively transferred to three non-transparent anaerobic bags (which not allowing any exchanges between inside and outside; Aluminum-plastic composite film, Hede, China) with 200 mL basal medium each and set as control groups; on the other hand, 2 mL freshly incubated strain zrk46 cells was respectively transferred into three dialysis bags (8,000–14,000 Da cutoff, which allowing the exchanges of substances smaller than 8,000 Da but preventing bacterial cells from entering or leaving the bag; Solarbio, China) with 200 mL basal medium each and set as experimental groups. In June 2021, all the samples were placed simultaneously in the deep-sea cold seep (where strain zrk46 was isolated) for 10 days during the cruise of *Kexue* vessel. After 10 days *in situ* cultivation, cells of strain zrk46 were immediately collected and kept in the −80°C freezer for future analysis. Before the proteomic sequencing analysis, the cells were checked by 16S rRNA gene sequencing to confirm the purity. The detailed protocol for proteomic sequencing was performed as described above.

#### Metatranscriptomic analysis of the metabolism of deep-sea SRB

To explore the actual metabolic characteristics of SRB conducted in the deep-sea cold seep, the *in situ* metatranscriptomic analysis was performed. Two cold seep sediment samples (TPK, cold seep vent area; TPKF, far away from cold seep vent area) were selected for metatranscriptomic sequencing analysis in Shanghai Biozeron Biothchnology Co., Ltd. (Shanghai, China). Total RNAS were extracted from these sediments using TRIzol Reagent according the manufacturer’s instructions and genomic DNA was removed using DNase I (TaKara, Japan). Then RNA quality was determined using 2100 Bioanalyser (Agilent, USA) and quantified using the ND-2000 (NanoDrop Technologies, USA). High-quality RNA sample (OD260/280 = 1.8–2.2, OD260/230 ≥ 2.0, RIN ≥6.5, 28S:18S ≥ 1.0, >10μg) is used to construct sequencing library. The detailed protocols were shown below: (1) Library preparation and Illumina Hiseq sequencing. Metatranscriptome libraries were prepared following TruSeq TM Stranded Total RNA Sample Preparation Kit from Illumina (San Diego, CA), using 5 μg of total RNA. Briefly, rRNA removal by Ribo-Zero TM rRNA Removal Kits from Illumina (San Diego, USA), fragmented using fragmentation buffer. cDNA synthesis, end repair, A-base addition, and ligation of the Illumina-indexed adaptors were performed according to Illumina’s protocol. Libraries were then size selected for cDNA target fragments of 200–300 bp on 2% Low Range Ultra Agarose followed by PCR amplified using Phusion DNA polymerase (NEB, USA) for 15 PCR cycles. Metatranscriptome sequencing was performed by Shanghai Biozeron Biothchnology Co., Ltd. (Shanghai, China). All samples were sequenced in the Illumina HiSeq 2500 instrument. Libraries were prepared with a fragment length of approximately 450 bp. Paired-end reads were generated with 150 bp in the forward and reverse directions. (2) Reads quality control and mapping. The raw paired end reads were trimmed and quality controlled by Trimmomatic with parameters (SLIDINGWINDOW:4:15 MINLEN:75) (version 0.36, http://www.usadellab.org/cms/uploads/supplementary/Trimmomatic). Then clean reads that aligned to the host genome were also removed. This set of high-quality reads was then used for further analysis. (3) Metatranscriptome Assembly and Annotation. The clean reads were aligned to the SILVA SSU (16S/18S) and SILVA LSU (23S/28S) databases in order to remove rRNA related reads using SortMeRNA (http://bioinfo.lifl.fr/RNA/sortmerna/) software. Then clean data from all samples were used to do assembly with megahit (http://www.l3-bioinfo.com/products/megahit.html). All the genes were predicted by METAProdigal (http://compbio.ornl.gov/prodigal/). Then non-redundant gene catalog were constructed with 95% identity and 90% coverage by CD-HIT (http://www.bioinformatics.org/cd-hit/). All genes searched against the NCBI protein nonredundant (NR), String, and KEGG databases using BLASTp to identify the proteins that had the highest sequence similarity with the given transcripts to retrieve their function annotations and a typical cut-off E-values less than 1.0 × 10^−5^ was set. BLAST2GO (http://www.blast2go.com/b2ghome) program was used to get GO annotations of unique assembled transcripts for describing biological processes, molecular functions and cellular components. Metabolic pathway analysis was performed using the Kyoto Encyclopedia of Genes and Genomes (KEGG, http://www.genome.jp/kegg/). (4) Differential expression analysis and functional enrichment. To identify DEGs (differential expression genes) between the two different samples, the expression level for each transcript was calculated using Salmon (https://github.com/COMBINE-lab/salmon). RSEM (http://deweylab.biostat.wisc.edu/rsem/) was used to quantify gene and isoform abundances. R statistical package software EdgeR (Empirical analysis of Digital Gene Expression in R, http://www.bioconductor.org/packages/2.12/bioc/html/edgeR.html) was utilized for differential expression analysis. The DEGs between two samples were selected using the following criteria: the logarithmic of fold change was greater than 1 and the false discovery rate (FDR) should be less than 0.05. To understand the functions of the differential expressed genes, GO functional enrichment and KEGG pathway analysis were carried out by Goatools (https://github.com/tanghaibao/Goatools) and KOBAS (http://kobas.cbi.pku.edu.cn/home.do) respectively. DEGs were significantly enriched in GO terms and metabolic pathways when their Bonferroni-corrected *p*-value was less than 0.05.

### Quantification and statistical analysis

All of the statistical details of experiments can be found in the figure legends. Statistical analysis was performed using GraphPad Prism 8.2.0.
