# Supplementary material for: Multi-omics analyses provide insights into the sulfur metabolism of a novel deep-sea sulfate-reducing bacterium
Source: iScience. 2024 May 23;27(6):110095. doi: 10.1016/j.isci.2024.110095 (PMC11214288; doi:10.1016/j.isci.2024.110095)
Supplement: Document S1. Figures S1 and S2 and Tables S1–S3 [file mmc1.pdf]

iScience, Volume *27*

Supplemental information

Multi-omics analyses provide insights into the sulfur metabolism of a novel deep-sea sulfate-reducing bacterium

Chong Wang, Rikuan Zheng, and Chaomin Sun

Supplementary Information

Multi-omics analyses provide insights into the sulfur metabolism of a novel deep-sea sulfate-reducing bacterium

Chong Wang[1,2,3], Rikuan Zheng[1,2,3], Chaomin Sun[1,2,3,4*]

[1]CAS and Shandong Province Key Laboratory of Experimental Marine Biology & Center of Deep Sea Research, Institute of Oceanology, Chinese Academy of Sciences, Qingdao, China.

[2]Laboratory for Marine Biology and Biotechnology, Qingdao National Laboratory for Marine Science and Technology, Qingdao, 266071, China

[3]Center of Ocean Mega-Science, Chinese Academy of Sciences, Qingdao, 266071, China

[4]College of Earth Science, University of Chinese Academy of Sciences, Beijing 100049, China

[*] Corresponding author

Chaomin Sun Tel.: +86 532 82898857; fax: +86 532 82898857.

E-mail address: sunchaomin@qdio.ac.cn

Supplementary Figures

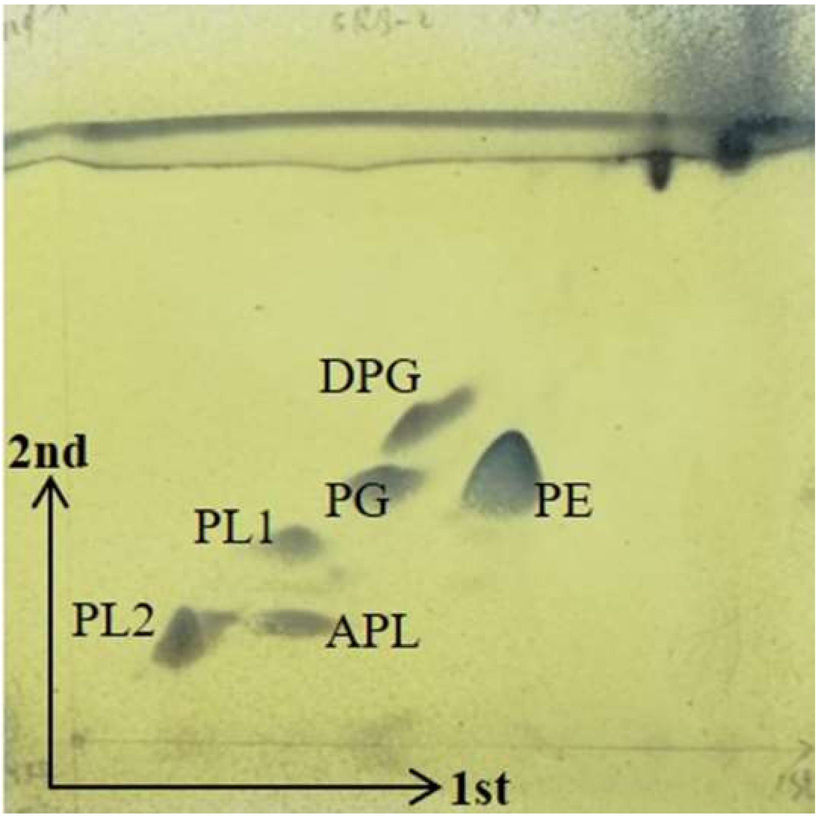

Figure S1. The polar lipids of strain zrk46 as revealed by two-dimensional TLC, related to Figure 2. Chloroform/methanol/water (65:25:4, v/v/v) was used in the first direction, followed by chloroform/methanol/acetic acid/water (80:12:15:4, v/v/v/v) in the second direction. The plate was sprayed with 10% ethanolic molybdophosphoric acid. Abbreviations: PE, phosphatidylethanolamine, DPG, diphosphatidylglycerol, PG, phosphatidylglycerol, APL, aminophospholipid, and PL, two phospholipids.

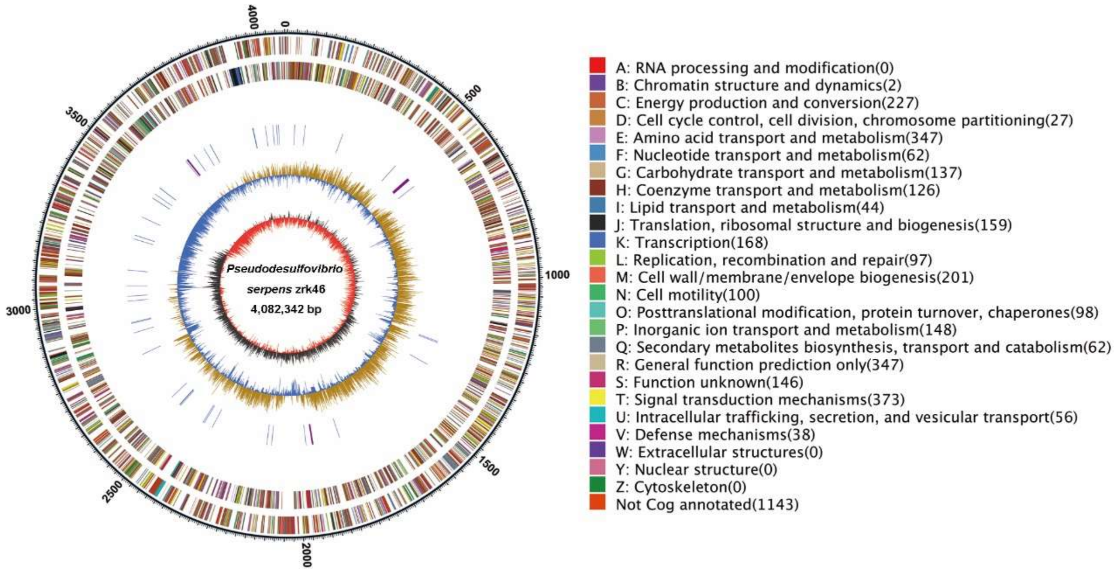

Figure S2. Circular diagram of the genome of strain zrk46, related to Figure 2. Rings indicate, from outside to the center: a genome-wide marker with a scale of 320 kb; forward strand genes, colored by COG category; reverse strand genes, colored by COG category; gene function annotation (COG, KEGG, GO, NR, CAZy, TCDB); RNA genes (tRNAs blue, rRNAs purple); GC content; GC skew. Different colors represent different COG functional classifications, shown on the panel right.

SUPPLEMENTARY TABLES

Table S1. Differential physiological characteristics of the novel strain zrk46 and the closely related type strain *Pseudodesulfovibrio profundus* strain DSM 11384^T. Strains: 1, zrk46 (all data from this study); 2, DSM 11384^T (all data from this study except DNA G+C content and polar lipids). +, Positive result or growth; -, negative result or no growth; NR, not reported.

Characteristic	1	2
Temperature range for growth (°C)	16-45	20-45
Optimum	28	28
pH range for growth	6.0-8.5	6.0-8.0
Optimum	7.0	7.0
NaCl range for growth (%)	0.5-8.0	2.0-10.0
Optimum	4.0	6.0
Oxidase activity	+	+
Catalase activity	+	+
Electron donors		
Fumarate	+	-
Acetate	+	+
Propionate	-	-
Butyrate	+	-
Methanol	-	-
Glycine	-	-
Ethanol	-	-
Formate	+	-
Lactate	+	+
Succinate	+	-
Malate	+	+
Electron acceptors		
Sulfate	+	+
Sulfite	+	-
Thiosulfate	+	-
Nitrate	+	-
Nitrite	-	-
Polar lipids	DPG, PE, 2PL, PG, APL	NR
Major fatty acids (>10 %)	iso-$C_{15:0}$, $C_{16:0}$	iso-$C_{15:0}$, anteiso-$C_{15:0}$
Isolation source	deep-sea cold seep sediment	surface seawater

Table S2. Comparison of the main fatty acids (%) of strain zrk46 with its closest relative *Pseudodesulfovibrio profundus* strain DSM 11384^T. Strains: 1, zrk46 (data from this study); 2, *Pseudodesulfovibrio profundus* strain DSM 11384^T (data from this study). Fatty acids amounting to <1 % in all strains are not shown. TR, Trace amount (<1 %).

**Fatty acid**	**Percentage (w/v) of total fatty acids**	
	**1**	**2**
$C_{14:0}$	1.61	2.02
iso-$C_{15:0}$	28.53	22.61
anteiso-$C_{15:0}$	9.66	15.22
iso-$C_{16:0}$	3.61	2.06
$C_{16:1}$*cis*9	6.17	2.25
$C_{16:0}$	17.29	7.50
iso-$C_{17:0}$	6.21	1.15
anteiso-$C_{17:0}$	1.46	TR
$C_{17:0}$	1.82	TR
$C_{18:1}$*cis*9	2.58	TR
$C_{18:1}$*cis*11/*trans*9/*trans*6	6.88	1.79
$C_{18:0}$	7.42	1.08

Table S3. Genomic characteristics of the whole-genome sequences of zrk46 and the most closely related strains. ANIb, ANIm, Tetra and *is*DDH values are presented with respect to the genome of the most closely related strains, *Pseudodesulfovibrio serpens* zrk46 as a reference.

Characteristics	zrk46	Aspo-2^{T}	J2^{T}	C1TLV30^{T}	DSM 11384^{T}
Gene Bank ID	CP051216	CP002431.1	CP014206.1	FO203427.1	LT907975.1
Genome size (bp)	4,082,342	3,629,109	3,966,573	3,646,098	4,217,908
No.scaffolds/contigs	1	1	1	1	3
GC-content (%)	53.26	62.60	63.50	49.90	52.80
ANIb (%)	100	72.36	72.83	71.47	73.31
ANIm (%)	100	83.43	83.63	83.85	83.87
Tetra	1	0.75847	0.73201	0.75404	0.93565
isDDH (%)	100	19.00	19.60	18.70	19.90
